# Irregular Bedtime and Nocturnal Cellular Phone Usage as Risk Factors for Being Involved in Bullying: A Cross-Sectional Survey of Japanese Adolescents

**DOI:** 10.1371/journal.pone.0045736

**Published:** 2012-09-19

**Authors:** Mamoru Tochigi, Atsushi Nishida, Shinji Shimodera, Norihito Oshima, Ken Inoue, Yuji Okazaki, Tsukasa Sasaki

**Affiliations:** 1 Department of Neuropsychiatry, Graduate School of Medicine, University of Tokyo, Tokyo, Japan; 2 Department of Schizophrenia Research, Tokyo Institute of Psychiatry, Tokyo, Japan; 3 Department of Neuropsychiatry, Kochi Medical School, Kochi, Japan; 4 Office for Mental Health Support, University of Tokyo, Tokyo, Japan; 5 Department of Public Health, Fujita Health University School of Medicine, Aichi, Japan; 6 Tokyo Metropolitan Matsuzawa Hospital, Tokyo, Japan; 7 Department of Health Education, Graduate School of Education and Office for Mental Health Support, University of Tokyo, Tokyo, Japan; Hungarian Academy of Sciences, Hungary

## Abstract

**Purpose:**

A number of studies have tried to identify risk factors for being involved in bullying in order to help developing preventive measures; however, to our knowledge, no study has investigated the effect of nocturnal lifestyle behavior such as sleep pattern or cellular phone usage. In the present study, we aimed to investigate the relationship between school bullying and sleep pattern or nocturnal cellular phone usage in adolescents. The effect of school size on school bullying was also examined.

**Methods:**

Data from the cross-sectional survey of psychopathologies conducted for 19,436 Japanese students from 45 public junior high schools (7^th^–9^th^ grade) and 28 senior high schools (10^th^–12^th^ grade) were analyzed.

**Results:**

Bullying status was significantly associated with irregular bedtime (OR = 1.23 and 1.41 for pure bullies and bully-victims, respectively) and e-mail exchange or calling after lights-out (OR = 1.53 and 1.31 for pure bullies and bully-victims, respectively) after controlling domestic violence and substance usage. In addition, school size was significantly associated with the increased risk of bullying in junior high school students (OR = 1.13 for bully-victims).

**Conclusions:**

The present results suggested that sleep pattern and nocturnal cellular phone usage might be risk factors for being involved in school bullying in adolescents. Although further accumulation of data is needed, progressive trend towards nocturnal lifestyle and increasing usage of cellular phone might impair the well-being of adolescents. School-based interventions for lifestyle including sleep pattern and cellular phone usage may be encouraged to reduce school bullying.

## Introduction

Bullying is defined as an aggressive behavior which occurs repeatedly in interpersonal relationship where power imbalance exists [1]. The recent follow-up survey in Japan showed that more than 90% of the students had the experience of being bullied during the period from 4^th^ to 9^th^ grade, which means bullying could be a stressful live event for most of Japanese adolescents [2]. Bully-related suicides in adolescents are becoming a serious social problem in Japan. Bullying can increase the risk for physical and psychosocial problems in all the participants, not only victims but also bullies and bully-victims [3]. A number of studies described that victims experience various kinds of internalizing problems including anxiety, depression, and low self-esteem [4], [5]. The victims may also be at an elevated risk of psychosis [6]. Among bullies, increased risk for externalizing problems, such as aggression and antisocial behaviors, has been reported [7]. Bully-victims, who are victimized by others and bully others, are considered to be a distinct group with the most severe problems [3]. A series of recent studies have suggested that bully-victims are high in levels of both aggression and depression, and low on measured scores for academic competence, prosocial behavior, self-control, social acceptance, and self-esteem [8]. Thus, bullying is an extremely important issue which could affect the well-being of adolescents. To help developing preventive measures, a number of studies have tried to identify risk factors for being involved in bullying. Most of the studies have focused on the individual characteristics of victims, such as physical aggression, internalizing problems, and behavioral regulation problems [9]–[12]. Socioenvironmental factors, such as family, school, and neighborhood, were also examined as the risk factors for involvement in bullying [13]–[16]; however, few studies have investigated the relationship between lifestyle behavior and involvement in bullying. Turagabeci et al. [17] showed that healthy lifestyle, including nutrition, hygiene practices, and physical activity, was associated with decreased risk for being bullied; however, to our knowledge, no study has investigated the effect of lifestyle behavior such as sleep pattern or cellular phone usage. Considering that nocturnal lifestyle and cellular phone usage are becoming more common among children, it may be worth examining their effect on the risk for being involved in bullying. In the present study, we aimed to investigate the association between school bullying and sleep pattern or nocturnal cellular phone usage by using data from the cross-sectional survey of 19,436 Japanese junior and senior high school students. The effect of school size, which was shown to be associated with an increased risk for being a bully-victim in Bowes et al. [16], was also examined.

## Subjects and Methods

### Subjects

We used data from the cross-sectional survey of psychopathologies conducted from 2008 to 2009 in Kochi and Mie prefectures, Japan. Both prefectures are located in the mid to west part of Japan and approximately 300 km apart from each other, including suburban areas centering on prefectural capitals (populations of prefectural capitals are approximately 340,000 and 290,000, for Kochi and Mie, respectively). In this survey, data were collected from students from 45 public junior high schools (7^th^–9^th^ grade) and 28 senior high schools (10^th^–12^th^ grade). The total number of the current students of those high schools was 19,436 at the survey. As a procedure, one of the authors approached the school principals about participation in the survey, and the principals then consulted with teachers and parents. In the participating schools, teachers handed self-report questionnaires and envelopes to their students. When they were distributed, the teachers explained to the students 1) that participation in the study was anonymous and voluntary, and 2) that strict confidentiality would be maintained. The students were requested to seal the completed questionnaire in the provided envelop. Research staff collected the sealed questionnaires at each school.

We complied with Japan's Ethical Guidelines for Epidemiological Research, and the study was approved by the ethics committees of the Tokyo Institute of Psychiatry, the Mie University School of Medicine, and the Kochi Medical School. According to the Japanese law, attendance to junior high school is compulsory, while attendance to senior high school is not.

### Measures

The questionnaire included items on 1) bullying and victimization, 2) the Japanese version of 12-item General Health Questionnaire (GHQ-12), and 3) other variables including demographic characteristics and lifestyle behaviors. An expert in child and adolescent psychology and three school teachers from the participating schools, including a Japanese language teacher, examined the questions for age-appropriateness and reading comprehension.

#### Bullying and victimization

Questions about the experience of being bullied or bullying others were included in the questionnaire. The items were 1) “Have you been bullied within the past year?”, and 2) “Have you bullied others within the past year?”, with a choice of two responses, ‘yes’ or ‘no’. Based on the responses, we classified the subjects into the following four groups: 1) those who had not bullied and not been victimized (uninvolved); 2) those who had bullied but not been victimized (pure bully); 3) those who had been victimized but not bullied others (pure victim); and 4) those who had both bullied and been victimized within the past year (bully-victim).

#### The General Health Questionnaire (GHQ-12)

The GHQ-12 is one of the most widely used self-report screening tools for non-psychotic psychiatric symptoms, particularly symptoms of anxiety or depression [18]. The validity and reliability of the Japanese version of the GHQ-12 have been confirmed [19]. The GHQ-12 was originally applied to adult populations and subsequently used and validated for younger populations [20]. A four-point scale with binary scoring (0011) was used for each of the questions. Responses of ‘1’ were then added together to form the total score, with a range from 0 (best possible) to 12 (worst possible).

#### Other variables

In our questionnaire, we asked the subjects about 1) nocturnal sleep duration, 2) irregular bedtime, 3) having a cellular phone, 4) e-mail exchange or calling after lights-out, 5) usage of recreational drugs (ever), 6) the experience of smoking (within a month), 7) the experience of drinking alcohol (within a month), and 8) the experience of domestic violence from adults (within a month). Item 2) was rated by a four-point scale, items 3), 4), and 8) were answered ‘yes’ or ‘no’, and items 5), 6), and 7) were rated ‘once or more’ or ‘not at all’. The questionnaire also included items about demographic variables including sex, age, and family structure.

### Statistical analysis

In order to investigate the relationship between sleep pattern or cellular phone usage and the bullying status, logistic regression analysis was conducted. Odds ratio for being pure bullies, pure victims or bully-victims compared to the uninvolved subjects was calculated by entering nocturnal sleep duration, irregular bedtime, having a cellular phone, and e-mail exchange/calling after lights-out as independent factors and sex, age, survey area, the number of parents living together, drinking alcohol, usage of recreational drugs, and domestic violence from adults as covariates (forced entry). Nocturnal sleep duration was categorized into three groups: short (less than six hours), average (between six and eight hours), and long sleep durations (more than eight hours). Those who answered less than four hours and more than 11 hours were excluded, due to the lack of reliability. Odds ratios for short and long sleep durations were calculated in comparison with average sleep duration. Six and eight hours corresponded to 15 and 85 percentiles of the population in this study. When analyzing the data of junior high school student, school size was also entered as an independent factor. School size was not entered as an independent factor in the analysis of all or senior high school students because senior high schools are hierarchized based on the academic performance in Japan and the effect of school size may be covered by the academic hierarchy among the 28 senior high schools. All statistical analyses were carried out by using PASW Statistics 18 (SPSS Inc., Chicago, IL).

## Results

**Figure 1:**
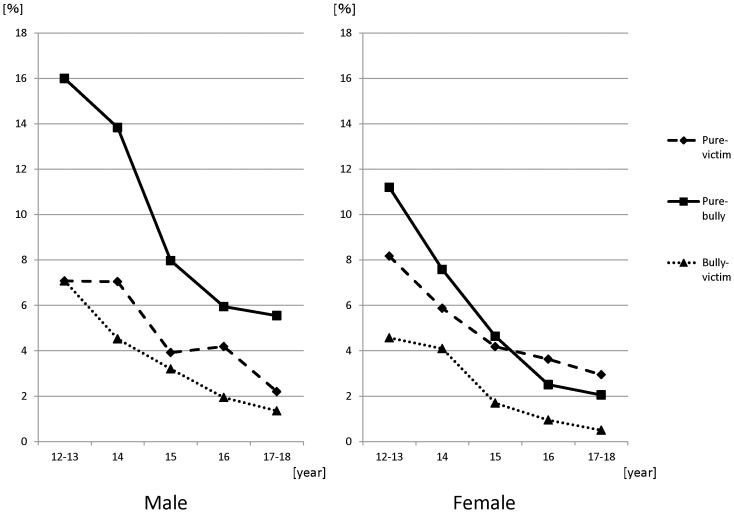
Changes of the prevalence of each bullying status by age. All three categories of the bullying status had a tendency of consistent decrease with age.

Out of 19,436 students of the 45 junior and 28 senior high schools, 798 students (4.1%) were absent on the days of the survey, and 388 students (2.0%) did not agree to participate in the study. Thus, the total of 18,250 students (93.9%) answered the questionnaire. Out of 18,250 subjects, 146 were excluded due to missing data for bullying status. Consequently, 18,104 subjects (8,992 males and 9,112 females, mean age  = 15.2 years (S.D. = 1.7)) were analyzed. Among them, 15,385 (85.1%) were uninvolved in the bullying problems, 1,346 (7.4%) were pure bullies, 859 (4.7%) were pure victims, and 514 (2.8%) were bully-victims. The demographics by the bullying status are summarized in Table 1. Changes of the prevalence of each bullying status by age are illustrated in Figure 1. All three categories of the bullying status had a tendency of consistent decrease with age. Regarding the experience of drinking alcohol, pure bullies (22.5%) and bully-victims (22.7%) showed larger frequencies compared to other two categories (12.5% and 13.4% for pure uninvolved subjects and pure victims, respectively). Having the experience of recreational drug usage was the most frequent in bully-victims (4.1%). Experience of domestic violence from adults was also the most frequent in bully-victims (17.0%), which was followed by pure victims and pure bullies (12.0% and 9.1%, respectively). Total GHQ-12 score was the highest in pure victims (mean  = 5.7 (S.D. = 3.5)) and bully-victims (mean  = 5.7 (S.D. = 3.2)), which was followed by pure bullies (mean  = 3.9 (S.D. = 3.2)). The difference between pure bullies and uninvolved subjects (mean  = 3.3 (S.D. = 3.0)) was relatively small.

**Table 1:** Demographics of the subjects*.

Number of Subjects	Uninvolved	Pure victim	Pure bully	Bully-victim
Total (7^th^–12th)	15385 (100)	859 (100)	1346 (100)	514 (100)
Junior high school (7th–9th)	6681 (43.4)	563 (65.5)	969 (72.0)	407 (79.2)
Senior high school (10th–12th)	8704 (56.6)	296 (34.5)	377 (28.0)	107 (20.8)
Male	7385 (48.0)	425 (49.5)	870 (64.6)	318 (61.9)
Female	8000 (52.0)	434 (50.5)	476 (35.4)	196 (38.1)
Age (year)	15.4 (1.7)	14.6 (1.6)	14.4 (1.6)	14.1 (1.5)
Living with Father	12267 (79.7)	651 (75.8)	1087 (80.8)	409 (79.6)
Living with Mother	14145 (91.9)	784 (91.3)	1263 (93.8)	470 (91.4)
Drinking alcohol (within a month)**	1901 (12.5)	114 (13.4)	297 (22.5)	114 (22.7)
Usage of recreational drugs (ever)**	73 (0.5)	11 (1.3)	23 (1.7)	21 (4.1)
Domestic violence from adults (within a month)**	376 (2.5)	103 (12.0)	122 (9.1)	87 (17.0)
GHQ-12 score	3.3 (3.0)	5.7 (3.5)	3.9 (3.2)	5.7 (3.2)

* The number of the participants and percentage (in brackets) are shown in each column, except for age and GHQ-12 score, in whose column mean and SD (in brackets) are described instead.

** The numbers of missing data for each variable are 256 for drinking alcohol, 126 for usage of recreational drugs, and 135 for violence from adults in the home.

Table 2 summarizes sleep pattern and nocturnal cellular phone usage in the present subjects by the bullying status. Associations of the factors with each bullying status, analyzed by logistic regression and compared to the uninvolved subjects, were also shown. In the analysis, factors including sex, age, survey area, the number of parents living together, drinking alcohol, usage of recreational drugs, and domestic violence from adults were adjusted. Nocturnal sleep duration was shorter in senior high school students than junior high school students through all categories. Associations of nocturnal sleep duration, as a whole, was statistically significant with pure victims in all students and junior high school students (Wald χ^2^ = 12.1 and 12.3, df = 2, p = 0.0023 and 0.0021, respectively, not shown in the table). Long sleep (>8 hrs) was significantly associated with pure victims in all students (Wald χ^2^ = 10.7, df = 1, p = 0.0011), and both short (<6 hrs) and long sleep (>8 hrs) were associated with pure victims in junior high school students (Wald χ^2^ = 5.93 and 7.82, df = 1, p = 0.015 and 0.0052, respectively). Long sleep (>8 hrs) was also significantly associated with bully-victims in all students and junior high school students (Wald χ^2^ = 4.02 and 4.87, df = 1, p = 0.045 and 0.027, respectively). Frequency of irregular bedtime was the highest in bully-victims, followed by pure bullies, pure victims, and uninvolved subjects in this order, both in junior and senior high school students. Irregular bedtime was significantly associated with pure bullies and bully-victims in all students and junior high school students (Wald χ^2^ = 6.72 and 7.81, df = 1, p = 0.0095 and 0.0052 in all students, and Wald χ^2^ = 5.37 and 5.76, df = 1, p = 0.021 and 0.016 in junior high school students, for pure bullies and bully-victims, respectively). In senior high school students, the same trends of the association were observed, while they were not statistically significant. Having a cellular phone and e-mail exchange/calling after lights-out were remarkably more frequent in senior high school students than in junior high school students. Having a cellular phone was significantly associated with pure victims in all students and pure bullies in junior high school students (Wald χ^2^ = 4.39 and 5.09, df = 1, p = 0.036 and 0.024, respectively). E-mail exchange/calling after lights-out was significantly associated with pure bullies in all, junior high, and senior high school students (Wald χ^2^ = 28.9, 12.6, and 17.6, df = 1, p = 7.75×10^−8^, 3.89×10^−4^, and 2.80×10^−5^, respectively), and bully-victims in all and senior high school students (Wald χ^2^ = 4.05 and 8.74, df = 1, p = 0.044 and 0.0031, respectively). School size was significantly associated with bully-victims in junior high school students (Wald χ^2^ = 6.23, df  = 1, p = 0.013).

**Table 2:** Relationship between sleep pattern, nocturnal cellular phone usage and the bullying status*.

	Uninvolved	Pure victim		Pure bully		Bully-victim	
	N (%)	N (%)	OR (95% CI)	N (%)	OR	N (%)	OR
***All (7^th^–12th)***							
Nocturnal sleep duration (hrs)	7.0 (1.1)	7.3 (1.3)		7.3 (1.2)		7.4 (1.2)	
Average sleep (6–8 hrs)	11853 (79.1)	579 (70.5)	Reference	988 (76.2)	Reference	350 (71.9)	Reference
Short sleep (<6 hrs)	1543 (10.3)	86 (10.5)	1.21 (0.95–1.55)	103 (7.9)	0.94 (0.75–1.18)	35 (7.2)	0.83 (0.57–1.23)
Long sleep (>8 hrs)	1591 (10.6)	156 (19.0)	1.40 (1.14–1.70) ¶	207 (15.9)	1.01 (0.85–1.20)	102 (20.9)	1.29 (1.01–1.65) ¶
Irregular bedtime (always)	2378 (15.5)	158 (18.4)	1.18 (0.97–1.44)	268 (20.0)	1.23 (1.05–1.44) ¶	126 (24.6)	1.41 (1.11–1.80) ¶¶
Having a cellular phone	11662 (76.5)	547 (64.0)	0.82 (0.69–0.99) ¶	920 (68.6)	1.13 (0.97–1.31)	313 (61.1)	0.92 (0.73–1.17)
E-mail exchange/calling**	3436 (22.6)	163 (19.1)	1.03 (0.84–1.25)	336 (25.1)	1.53 (1.31–1.79) †	104 (20.4)	1.31 (1.01–1.71) ¶
***Junior high school (7^th^–9th)***							
Nocturnal sleep duration (hrs)	7.4 (1.1)	7.5 (1.2)		7.5 (1.2)		7.5 (1.2)	
Average sleep (6–8 hrs)	5013 (77.1)	365 (67.6)	Reference	694 (73.8)	Reference	271 (69.8)	Reference
Short sleep (<6 hrs)	363 (5.6)	45 (8.3)	1.55 (1.09–2.19) ¶	59 (6.3)	1.08 (0.79–1.46)	21 (5.4)	0.84 (0.51–1.40)
Long sleep (>8 hrs)	1124 (17.3)	130 (24.1)	1.38 (1.10–1.72) ¶¶	187 (19.9)	1.03 (0.86–1.24)	96 (24.7)	1.34 (1.03–1.74) ¶
Irregular bedtime (always)	956 (14.4)	96 (17.1)	1.16 (0.91–1.49)	185 (19.2)	1.25 (1.04–1.51) ¶	94 (23.2)	1.41 (1.06–1.86) ¶
Having a cellular phone	3616 (54.8)	281 (50.2)	0.89 (0.73–1.10)	566 (58.6)	1.21 (1.03–1.43) ¶	213 (52.6)	0.99 (0.78–1.28)
E-mail exchange/calling **	874 (13.3)	72 (12.9)	0.94 (0.69–1.27)	177 (18.3)	1.46 (1.18–1.80) †	58 (14.4)	1.06 (0.75–1.50)
School size (per 100 students)	349.5 (162.0)	350.3 (156.1)	1.01 (0.94–1.09)	375.1 (151.6)	1.06 (0.99–1.13)	377.0 (154.8)	1.13 (1.03–1.25) ¶
***Senior high school (10^th^–12th)***							
Nocturnal sleep duration (hrs)	6.8 (1.1)	6.9 (1.2)		6.7 (1.1)		6.9 (1.2)	
Average sleep (6–8 hrs)	6840 (80.6)	214 (76.2)	Reference	294 (82.1)	Reference	79 (79.8)	Reference
Short sleep (<6 hrs)	1180 (13.9)	41 (14.6)	0.99 (0.69–1.41)	44 (12.3)	0.81 (0.57–1.14)	14 (14.1)	0.86 (0.47–1.59)
Long sleep (>8 hrs)	467 (5.5)	26 (9.3)	1.53 (0.98–2.39)	20 (5.6)	0.94 (0.58–1.51)	6 (6.1)	1.01 (0.43–2.37)
Irregular bedtime (always)	1422 (16.4)	62 (20.9)	1.20 (0.88–1.65)	83 (22.1)	1.21 (0.91–1.60)	32 (29.9)	1.43 (0.88–2.34)
Having a cellular phone	8046 (93.1)	266 (90.2)	0.79 (0.50–1.23)	354 (94.1)	1.19 (0.72–1.96)	100 (93.5)	1.11 (0.44–2.84)
E-mail exchange/calling**	2562 (29.7)	91 (31.1)	1.08 (0.82–1.42)	159 (42.4)	1.66 (1.31–2.10) †	46 (43.4)	1.93 (1.25–2.98) ¶¶

* The number of the participants and percentage (in brackets) are described in each column, except for nocturnal sleep duration and school size, in whose column mean and SD (in brackets) are described instead. The numbers of missing data for each variable are 511 for sleeping hours, 45 for irregular bedtime, 157 for having a mobile phone, and 184 for e-mail or calling after bedtime. The odds ratios compared to the uninvolved subjects were calculated by logistic regression analysis adjusted for sex, age, survey area, the number of parents living together, drinking alcohol, usage of recreational drugs, and domestic violence from adults (forced entry).

** E-mail exchange or calling after lights-off (everyday)

¶; p<0.05, ¶¶; p<0.01, †; p<0.0001

## Discussion

The present study investigated the relationship between school bullying and sleep pattern or nocturnal cellular phone usage in the Japanese adolescents (7^th^–9^th^ grade). Bullying behavior was significantly associated with irregular sleep and e-mail exchange/calling after lights-out after controlling sex, age, survey area, the number of parents living together, drinking alcohol, usage of recreational drugs, and domestic violence from adults. In addition, school size was also significantly associated with the increased risk of bullying in junior high school students (7^th^–9^th^ grade). To our knowledge, this is the first study to observe the significant effect of nocturnal lifestyle such as sleep pattern and cellular phone usage on school bullying.

The association between irregular bedtime and the bullying status suggests that problems in sleep pattern may be a causing or moderating factor for bullying behavior. In junior high school students, irregular bedtime was significantly associated with both being pure bully and bully-victim. The association was not statistically significant in senior high school students, while the trend was the same as in junior high school students when the odds ratios were referred. The statistical difference between junior and senior highs school may be explicable by the statistical power because the number of subjects and the prevalence of bullying were smaller in senior high school than in junior high school. Adolescence is a developmental period accompanied with changes in sleep characteristics [21]. It has been suggested that the way of sleeping critically affect daytime functioning, including thinking, feeling, and behavior, in adolescence [22]–[24]. Wake-up and fall-asleep rhythm is spontaneously accompanied with rise and decline rhythm of the body temperature [25]. When this reciprocal interaction is impaired, physical or mood disturbance occurs and may work as a biological mechanism for the critical effect of the problematic sleep pattern on bullying behavior [26]. The present result may warrant the importance of school-based interventions to improve nocturnal behaviors as a method to prevent school bullying.

E–mail exchange/calling after lights-out was remarkably associated with pure bullies in both junior and senior high school students, and also with bully-victims in senior high school students. The associations suggest that having a cellular phone and e-mail exchange/calling after lights-out might facilitate bullying. For instance, a cellular phone may work as a major communication method to conduct psychological bullying including excluding, isolating, and gossiping. It may be necessary to increase awareness of electronic bullying among students, parents, and school personnel for prevention. Nocturnal sleep duration was also associated with the bullying status, including being pure victim and being bully-victim, especially in junior high school students. This could be due to insomnia or hypersomnia as a symptom of depression resulting from victimization.

It is of note that a significant association was observed between the bullying status and school size. Large school size was significantly associated with the bully-victim status. Bowes et al. [16] showed that large school size was associated with an increased risk for victimization in seven-year-old children. The possible explanation for the effect of school size is that escaping from teachers' supervision might be relatively easier for students in large-size schools. This finding may be valuable from a viewpoint of developing preventive measures, while further research is needed to confirm the result and elucidate the mechanism.

Prevalence of the bullying status was basically comparable with previous Japanese studies for junior high school students (12%, 6%, and 5% in the present study vs. 9%, 6%, and 5%, and 14%, 7%, and 5% in 1,994 and 1,248 students in the previous studies [27], [28], for pure bullies, pure victims, and bully-victims, respectively). No previous data was available for senior high school students in Japan. GHQ-12 score, which could represent increased internalizing problems, i.e. anxiety and depression [4], was elevated in pure victims. Drinking alcohol and recreational drug usage were in contrast, increased in pure bullies, which might correspond to externalizing problems, such as aggression and antisocial behaviors [7]. The risk for these problems was the highest in bully-victims, which supports the previous finding that they are a distinctive group of the most troubled adolescents [3].

Caution may however be needed in interpretation of the present data. First, this is a cross-sectional survey, and therefore there is a possibility that the problematic sleep pattern and/or e-mail exchange/calling after lights-out might be a result, not a cause, of the bullying status. A longitudinal study is needed for the answer to the question. Second, data on socioeconomic status was not available and therefore not controlled. Third, answers from absent students were not available, while bullying and victimization could be more prevalent among the frequent or long-term absentees. Lastly, we used simple self-report questions with a relatively small number of items to evaluate the bullying and victimization. Subjects could over- or under-report on these topics. More details of sleep pattern and problematic cellular phone usage, such as differences between weekdays and weekends, may be examined in future studies.

In conclusion, the present study suggested that irregular bedtime and cellular phone usage after lights-out might be risk factors for being involved in school bullying in adolescents. Progressive trend towards nocturnal lifestyle and increasing usage of cellular phone might impair the well-being of adolescents. School-based interventions for lifestyle including sleep pattern and cellular phone usage may be encouraged to reduce school bullying.
